# Research and implementation of a whole-genome sequencing surveillance system for outbreak detection

**DOI:** 10.1017/ash.2022.211

**Published:** 2022-05-16

**Authors:** Alexander Sundermann, Marissa Griffith, Vatsala Rangachar Srinivasa, Kady Waggle, Melissa Saul, Ashley Ayres, Graham Snyder, Jane Marsh, Lee Harrison

## Abstract

**Background:** Traditional infection prevention (IP) methods for outbreak detection often rely on geotemporal clustering confined to single locations. We recently developed the Enhanced Detection System for Healthcare-Associated Transmission (EDS-HAT), which combines whole-genome sequencing (WGS) surveillance and machine learning of the electronic health record (EHR). Our retrospective research findings show potential transmissions averted and cost savings using EDS-HAT in real time. Here, we describe the process and initial findings from EDS-HAT real-time implementation. **Methods:** Real-time whole-genome sequencing surveillance began on November 1, 2021. Patient cultures positive for select bacterial pathogens who were hospitalized for ≥3 days or had a recent healthcare exposure in the prior 30-days were collected. Isolates were deemed genetically related if ≤15 single-nucleotide polymorphisms (SNPs) were identified for all organisms except *Clostridioides difficile* (≤2 SNPs). Clusters were manually investigated by both research and IP teams, and interventions were performed by the IP team. Data on collection, analysis, notification, and intervention dates were gathered. **Results**: As of January 11, 2022, 413 isolates had undergone whole-genome sequencing. Among them, 18 unique patient isolates were genetically related to ≥1 other isolate, comprising 7 clusters (range, 2–6 patients). Notable findings include a *Pseudomonas aeruginosa* cluster possibly related to a shared bronchoscope, a pseudo-outbreak of *Serratia marcescens* related to autopsy blood culture practice, and a cluster of vancomycin-resistant *Enterococcus faecium* on a shared transplant unit. Only 1 cluster of 2 isolates of *Klebsiella pneumoniae* had no known possible transmission routes. The median turnaround time from patient’s culture date to IP notification was 19 days (range, 13–28), with noted delays over the winter holiday. **Concusions:** Real-time WGS can identify small clusters including potentially interruptible transmission routes. Rapid turnaround time, coordination between clinical and genomic laboratories, and a robust IP team are key factors in implementing a WGS surveillance program. Real-time WGS surveillance has the potential to reduce costs for hospitals, improve patient safety, and save lives.

**Funding:** None

**Disclosures:** None

